# Porcine Deltacoronavirus Infection and Transmission in Poultry, United States^1^

**DOI:** 10.3201/eid2602.190346

**Published:** 2020-02

**Authors:** Patricia A. Boley, Moyasar A. Alhamo, Geoffrey Lossie, Kush Kumar Yadav, Marcia Vasquez-Lee, Linda J. Saif, Scott P. Kenney

**Affiliations:** The Ohio State University, Wooster, Ohio, USA (P.A. Boley, M.A. Alhamo, K.K. Yadav, M. Vasquez-Lee, L.J. Saif, S.P. Kenney);; The Ohio State University, Columbus, Ohio, USA (G. Lossie)

**Keywords:** Porcine deltacoronavirus, swine disease, interspecies transmission, chickens, turkeys, poultry, zoonoes, viruses, coronaviruses, United States

## Abstract

Coronaviruses cause respiratory and gastrointestinal diseases in diverse host species. Deltacoronaviruses (DCoVs) have been identified in various songbird species and in leopard cats in China. In 2009, porcine deltacoronavirus (PDCoV) was detected in fecal samples from pigs in Asia, but its etiologic role was not identified until 2014, when it caused major diarrhea outbreaks in swine in the United States. Studies have shown that PDCoV uses a conserved region of the aminopeptidase N protein to infect cell lines derived from multiple species, including humans, pigs, and chickens. Because PDCoV is a potential zoonotic pathogen, investigations of its prevalence in humans and its contribution to human disease continue. We report experimental PDCoV infection and subsequent transmission among poultry. In PDCoV-inoculated chicks and turkey poults, we observed diarrhea, persistent viral RNA titers from cloacal and tracheal samples, PDCoV-specific serum IgY antibody responses, and antigen-positive cells from intestines.

Coronaviruses (CoVs) cause respiratory and gastrointestinal disease in humans, poultry, swine, and cattle. CoVs (family *Nidovirales*, subfamily *Coronaviridae*, subfamily *Coronavirinae*) are composed of 4 genera, *Alphacoronavirus*, *Betacoronavirus*, *Gammacoronavirus*, and *Deltacoronavirus* (*1*). Viruses from each CoV genus have been detected in diverse host species, but gammacoronaviruses and deltacoronaviruses (DCoVs) have been isolated primarily in birds (*2*). Two members of the *Gammacoronavirus* genus, transmissible gastroenteritis coronavirus (TGEV) and porcine epidemic diarrhea virus (PEDV), cause severe diarrhea in swine. TGEV and PEDV infections have caused severe economic losses in many countries, including the United States (*3*–*5*). The *Betacoronavirus* genus includes the notable human pathogens OC43, HKU1, severe acute respiratory syndrome (SARS) CoV, and Middle East respiratory syndrome (MERS) CoV, which mostly cause respiratory symptoms (*6*–*9*). *Gammacoronavirus* includes avian enteric coronavirus and infectious bronchitis virus that mainly infect avian species (*10*). DCoVs previously were identified primarily in multiple songbird species and in leopard cats (*Prionailurus bengalensis*) (*11*).

Porcine deltacoronavirus (PDCoV) was initially detected in 2009 in fecal samples from pigs in Asia, but its etiologic role was not identified until 2014, when it caused diarrhea in pigs in the United States (*11*,*12*). The origin of PDCoV is unknown, but because of the widespread prevalence of DCoV in songbird species and genomic similarities, researchers suspect that PDCoV may have originated from an ancestral avian DCoV (*11*). 

In experiments, PDCoV caused diarrhea and gut lesions in infected piglets (*13*,*14*). It was detected in pigs in Hong Kong in 2012 (*11*) and the United States in February 2014 (*15**–**17*). Although not considered as deadly to pigs as PEDV (*13*,*18*), PDCoV continues to circulate and cause illness in swine herds worldwide. How PDCoV emerged in swine and how it spreads remain unknown.

In vitro studies have shown that PDCoV utilizes a conserved region of the protein aminopeptidase N (APN) gene to infect cell lines derived from multiple species, including humans, pigs, and chickens (*19*). As a potentially emerging zoonotic pathogen, studies of PDCoV prevalence in humans and its contribution to human disease are ongoing. The ability of PDCoV to infect cells of multiple species and cause illness and death in pigs makes it a priority pathogen that should be studied further.

In vivo studies could validate cell culture susceptibility findings and determine whether PDCoV causes infection and disease in species other than pigs. Virus cross-species transmission among hosts plays a major role in the evolution and diversification of viruses, appearing in many instances to be preferential to coevolving within an initial host (*20*). CoVs already have demonstrated a propensity for crossing species barriers, both in animal-to-animal spread and animal-to-human spread. Initial evidence of CoVs jumping from mammalian to avian species was reported from bovine CoV infecting turkeys but not chickens (*21*,*22*). As a zoonotic CoV transmission, SARS-CoV is believed to have jumped from bats or palm civets (*Paguma larvata*) to humans in 2002, causing 8,098 cases in 37 countries and 774 deaths (*11*). MERS-CoV emerged more recently, jumping from dromedary camels (*Camelus dromedarius*) to humans, and has caused 1,879 cases of respiratory illness in humans and 666 deaths (*10*). 

Understanding how cross-species transmission of CoVs occurs is critical to our ability to predict which viruses might be on the verge of SARS- or MERS-like pandemics. In addition, studies of CoV cross-species transmission can inform development of novel therapeutics and strategies to combat CoVs in susceptible animal hosts before they pose an imminent human health threat. We conducted experiments to determine the prevalence of PDCoV infection in and transmissibility among poultry.

## Materials and Methods

### Animals

We obtained 25 fourteen-day-old chickens (*Gallus gallus domesticus*) and 25 fourteen-day-old turkey poults (*Meleagris gallopavo*) from the specific pathogen–free flock of the Ohio Agricultural Research and Development Center of The Ohio State University (Wooster, Ohio, USA). This flock has no prior exposure to swine or to PDCoV, PEDV, or TGEV. After acclimating in Biosafety Level 2 (BSL-2) facilities for 1 day, all birds appeared healthy with no evidence of diarrhea or other clinical signs. Animal protocols used in this study were approved by the Institutional Laboratory Animal Care and Use Committee of The Ohio State University.

### Titration of PDCoV 

We determined PDCoV titers from intestinal contents of pigs by 50% tissue culture infectious dose (TCID_50_) assay (*23*). We seeded LLC porcine kidney (LLC-PK) cells at 5 × 10^4^ cells/well in a 96-well plate (BD Biosciences, https://www.bdbiosciences.com). We washed 100% confluent monolayers once with 200 µL of maintenance media (MM): minimal essential media (MEM), 4-(2-hydroxyethyl)-1-piperazineethanesulfonic acid (HEPES), GlutaMAX (GIBCO, https://www.thermofisher.com) consisting of MEM with 1% antibiotic-antimycotic solution, 1% nonessential amino acids, and 1% HEPES. We inoculated 100 µL from 10-fold dilutions of PDCoV in 8 replicates per dilution. Each plate included 1 row of negative control MM only with 5 µg/mL of Trypsin (Corning, https://www.corning.com). After absorption for 1 h, we added another 100 µL of MM with 5 µg/mL of Trypsin to each well. We monitored cytopathic effects for 3–7 days, calculated virus titers after immunofluorescent (IF) staining by using the Reed-Muench method (*24*), and expressed results as log_10_ TCID_50_/mL (*23*).

### Study Design

Birds were floor housed in a temperature-controlled BSL-2 containment room with wood litter shavings and provided ad libitum access to food and water. In consecutive experiments of poults and chicks, we randomly divided the flock of 25 birds into 2 groups, 15 uninfected and 10 infected birds. Each group was housed separately and inoculated through the choanal cleft. The uninfected group was inoculated with 200 µL of unfiltered, undiluted small intestine contents (SIC) from an uninfected gnotobiotic pig (GP-8). The infected group was inoculated with SIC from a PDCoV-infected pig (DC175) with 6.87 log_10_ TCID_50_/mL. One poult in the uninfected group died of unknown causes unrelated to known pathogens before inoculation.

After inoculation, we observed chicks and poults for clinical signs 2 times each day. We scored fecal consistency as follows: 0, solid; 1, pasty; 2, semiliquid; and 3, liquid (*25*). We considered a fecal consistency score of >2 as diarrhea. At 2 days postinoculation (dpi), we randomly assigned 5 birds from each uninfected group as sentinels and allowed them to comingle with the infected group for the duration of the experiment. We recorded body weights and collected cloacal swab, tracheal swab, and serum samples at 2, 4, 7, 9, 11, and 14 dpi. Except for sentinel birds, we euthanized 2 chicks and 2 poults from each group at 3 and 7 dpi for blood and tissue collection. We concluded the study at 14 dpi and euthanized the remaining 33 birds, including sentinels, for blood and tissue collection.

### Serum Antibody Detection

We modified and optimized an ELISA (*26*) to detect PDCoV-specific IgY antibodies in serum from PDCoV-inoculated chicks and poults. We added 50 µL of serum diluted 1:1,000 to the PDCoV antigen-coated and mock antigen-coated wells and incubated for 90 m at 37°C, then added 100 µL of biotin-conjugated antichicken IgY (Invitrogen Goat anti-Chicken IgY [H+L] Secondary Antibody, Biotin; ThermoFisher, https://www.thermofisher.com) or biotin-conjugated antiturkey IgY (Goat Anti-Turkey IgY (H+L) Biotin pAb; Cell Sciences, https://www.cellsciences.com) at a dilution of 1:10,000 and incubated at 37°C for 1 h. We added 100 µL of HRP-Conjugated Streptavidin (ThermoFisher) to each well at a dilution of 1:5,000 and incubated at 37°C for 1 h. We washed wells with phosphate buffered saline solution with 0.05% Tween-20 (×5) between each step. We added 3,3′,5,5′-tetramethylbenzidine substrate (SureBlue TMB 1-Component Microwell Peroxidase Substrate; Seracare, https://www.seracare.com), then added 100 µL of 0.3 mol/L sulfuric acid to stop the reaction. We read plates at an absorbance of 450 nm by using a SpectraMax F5 (Molecular Devices, https://www.moleculardevices.com) plate reader. We conducted statistical analysis by using Prism software (GraphPad, https://www.graphpad.com). We used analysis of variance to compare multiple groups and a 1-tailed Student *t*-test to compare groups of 2.

### Histopathology and IF Staining 

We examined gross tissues from small intestines, duodenum to ileum, and large intestines, cecum and colon, as well as other organs, including bursa, lung, liver, kidney, proventriculus, and spleen, and then fixed tissues in 10% neutral formalin for 1–2 days at room temperature for histopathology (*27*). We embedded, sectioned, and then stained samples with hematoxylin and eosin for light microscopy examination. We measured mean jejunal or ileal ratios of villus height and crypt depth (VH:CD) by using MetaMorph software (MetaMorph, Inc., https://www.metamorphsoftware.com), as described previously (*18*). We tested prepared tissues by IF staining to detect PDCoV antigen using a polyclonal rabbit antiserum against PDCoV (provided by E. Nelson, South Dakota State University, Brookings, SD, USA) (*28*). We also tested tissues from a PDCoV-infected pig for comparison.

### Real-Time Reverse Transcription-PCR 

We suspended cloacal swabs, tracheal swabs, SIC, and large intestine contents (LIC) in 1–4 mL MEM as a 10% suspension. We extracted RNA by using GenCatch Viral RNA Miniprep Kit (Epoch Life Science, https://www.fishersci.com). We further processed samples containing fecal matter by using OneStep PCR Inhibitor Removal Kit (Zymo Research Corporation, https://www.zymoresearch.com). 

We determined viral RNA titers by real-time reverse transcription-PCR (rRT-PCR), as reported previously (*23*). In brief, we amplified a 541-bp fragment of the M gene that covered the quantitative RT-PCR–amplified fragment. We designed 5′-CGCGTAATCGTGTGATCTATGT-3′ and 5′-CCGGCCTTTGAAGTGGTTAT-3′ primers according to the sequence of a strain from the United States, Illinois121/2014 (GenBank accession no. KJ481931). We purified the PCR products by using a QIAquick PCR Purification Kit (QIAGEN Inc., https://www.qiagen.com), sequenced, and then used these as the template to construct a quantitative RT-PCR standard curve. The detection limit of the rRT-PCR was 10 genomic equivalents (GEs)/reaction, which corresponded to 4.6 log_10_ GE/mL of PDCoV in cloacal and tracheal samples.

## Results

### Clinical Signs

By 2 dpi, 70% of infected chicks had diarrhea and fecal scores >2; that percentage decreased to 17% by 9 dpi (Table 1). By 14 dpi, most (5/6) of the remaining infected chicks had normal feces. The 5 uninfected sentinel chicks that comingled with the infected birds at 2 dpi demonstrated mild to moderate diarrhea 2 days after comingling (4 dpi); diarrhea peaked 5 days after comingling (7 dpi). At 14 dpi, only 2 sentinel chicks had abnormal feces. Two chicks in the uninfected group had transient diarrhea during the study but tested negative for known pathogens, including PDCoV.

**Table 1:** Prevalence of diarrhea in uninfected birds and birds experimentally inoculated with porcine deltacoronavirus*

Group	Days postinoculation					
2	4	7	9	11	14

Birds, total†	n = 49	n = 41	n = 41	n = 33	n = 33	n = 33
Chicks, n = 25						
Uninfected	0/15	1/8‡	2/8‡	1/6‡	0/6	0/6
Infected	7/10	5/8	5/8	1/6	0/6	1/6
Sentinel§	NA	1/5	3/5	1/5	0/5	0/5
Poults, n = 25						
Uninfected	0/14¶	0/7	0/7	0/5	0/5	0/5
Infected	5/10	2/8	6/8	5/6	4/6	5/6
Sentinel§	NA	0/5	1/5	4/5	5/5	5/5

*Uninfected birds were inoculated with small intestine contents from uninfected gnotobiotic pig. Fecal consistency scored on a scale of 0–3: 0, solid; 1, pasty; 2, semiliquid; 3. liquid. Values expressed as number of birds with a cloacal swab score of ≥2/total number of birds in the room on the indicated day postinoculation (dpi). NA, not applicable. †Two birds from each group, excluding sentinels, were randomly removed for necropsy at 3 dpi and 7 dpi .  ‡One chick in uninfected group consistently had a score of 3 for 7 d, another briefly scored a 2 at 7 dpi; both tested negative for porcine deltacoronavirus, *Salmonella*, and for parasites via fecal float analysis. Cause of diarrhea was unknown**.** §We removed 5 uninfected birds as sentinels and allowed them to comingle with infected birds at 2 dpi for the duration of the experiment. ¶One poult in uninfected group died before inoculation.

In poults, 50% exhibited diarrhea at 2 dpi. During the study, the rate of diarrhea increased, and poults did not recover by 14 dpi. (Table 1). Sentinel poults began exhibiting mild to moderate diarrhea 5 days after comingling (7 dpi), and by 9 dpi, 60% were affected. At 14 dpi, all 5 sentinel poults had moderate diarrhea. 

Two infected chicks necropsied at 3 dpi had distended gastrointestinal tracts containing a mixture of yellow liquid and gas. Similar but less extensive findings were seen in infected chicks at 7 dpi. No gross pathology was detected in infected chicks or sentinel chicks at 14 dpi. In necropsies of infected poults, we observed distended gastrointestinal tracts containing a mixture of yellow liquid and gas at all time points.

### Weights

**Figure 1:**
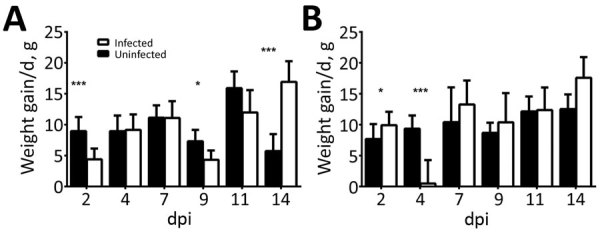
Average weight gain/day of (A) chicks and (B) turkey poults in a study of infection and transmission of porcine deltacoronavirus in poultry. Weights were taken at 2, 4, 7, 9, 11, and 14 dpi and differences were averaged by the number of days between time points. Weights for sentinel birds are excluded after 2 dpi. Error bars indicate upper half of SD. Statistically significant values are indicated: *p<0.05; **p<0.01; ***p<0.001. dpi, days postinoculation.

Birds were weighed before inoculation and then at 2, 4, 7, 9, 11, and 14 dpi. PDCoV infection greatly affected the chicks’ weight at 2 dpi (Figure 1, panel A). At 4 and 7 dpi, weight gain averages in infected chicks were comparable to those in uninfected birds, but at 9 and 11 dpi, infected chicks had gained much less weight than the uninfected chicks. By 14 dpi, infected chicks rebounded and showed compensatory weight gain at higher levels than uninfected chicks.

Poult weight gain responses differed from those of the chicks. At 2 dpi, infected poults were gaining weight at a much higher rate than uninfected poults (Figure 1, panel B). However, by 4 dpi, poult weight gain was severely curtailed; several lost weight, and the average weight gain for the infected group was 0.5 g, compared with almost 10 g for uninfected poults. By 7 dpi, the infected poults recovered and gained weight at a slightly higher, but not statistically significantly different, rate than the uninfected poults. This trend continued until the end of the study.

### Histopathology and IF Staining 

We examined tissue sections by using light microscopy. We noted suspect zymogen depletion in several poults in both the infected and uninfected groups, suggesting possible inanition. We conducted VH:CD measurements of the ileum and jejunum of intestinal tissues from chicks at 14 dpi (Table 2). Infected chicks had a VH:CD ratio of 4.26:1 compared with a ratio of 6.15:1 for uninfected chicks. The VH:CD ratio was lower in sentinel chicks than in uninfected chicks but the difference was not statistically significant. 

**Table 2:** Effect of porcine deltacoronavirus on villus height:crypt depth ratios of ileum or jejunum in experimental chicks*

Characteristics	Uninfected, n = 6	Infected, n = 6	Sentinel, n = 5
Villous height	370	353	547
Crypt depth	64	84	94
Ratio of villous height to crypt depth	6.15	**4.26**	5.87

*Values are expressed in millimeters and represent the average of <10 measurements/bird at 14 d postinoculation with porcine deltacoronavirus. Bold text indicates statistically significant difference, p = 0.04.

**Figure 2:**
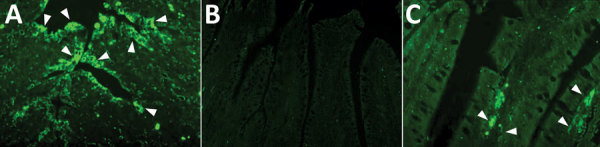
Detection of porcine deltacoronavirus (PDCoV) antigen in poultry by immunofluorescent (IF) staining in the intestines of poults inoculated with intestinal contents of a PDCoV-infected GF pig (DC175) (6.87 log_10_ 50% tissue culture infectious dose/mL) or a mock inoculate in a study of infection and transmission of porcine deltacoronavirus in poultry. A) PDCoV-infected pig intestine used as positive control; white arrows indicate widespread antibody staining. B) IF-stained jejunum of a poult (no. 42) at 14 dpi with no antigen-positive cells. C) IF-stained jejunum of a poult (no. 63) at 14 days postinoculation (dpi); white arrows indicate several PDCoV antigen-positive cells in the villous epithelial cells. Original magnification ×300.

We could not obtain enough measurements for poult tissues to provide accurate comparisons. IF tissue staining in infected poults demonstrated PDCoV antigen detectable in the epithelial cells lining the villi of the jejunum, although at reduced levels from the ileum and from infected porcine tissue (Figure 2, panel A). We also detected PDCoV antigen in numerous epithelial cells that had sloughed off and remained in the lumen of infected poults when compared with stained tissue sections from uninfected poults (Figure 2, panel B and C). We were unable to visualize a signal in tissues from chicks.

### Serum IgY Antibody Responses

We analyzed serum samples collected at 2, 4, 7, 9, 11, and 14 dpi. We used indirect ELISA to test samples at 2 and 14 dpi for PDCoV-specific IgY antibodies in all birds from each group, including sentinel birds. We assigned experimental values by averaging 3 replicates. Because we did not have positive controls in chicks and poults, we established a cutoff by using the average final optical density value of uninfected birds at 2 dpi plus 2 SD. We established a separate cutoff value for sentinel birds.

**Figure 3:**
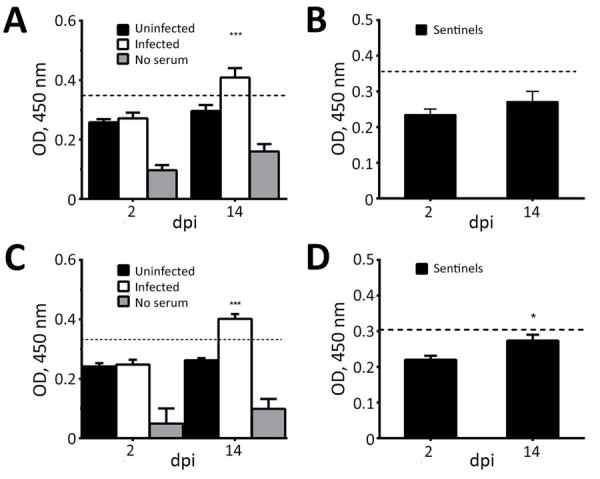
Detection of porcine deltacoronavirus (PDCoV)–specific IgY antibody titers in serum collected from chicks and turkey poults in a study of infection and transmission of porcine deltacoronavirus in poultry. A) Uninfected (n = 6) and infected chicks (n = 6). B) Sentinel chicks (n = 5). C) Uninfected (n = 5) and infected poults (n = 6). D) Sentinel poults (n = 5). OD values are blanked against a control of uncoated wells (carbonate buffer only). Values represent the average of 3 replicates; error bars represent upper half of SEM. Dashed line indicates cutoff, which was determined by using the mean of uninfected birds at 2 dpi +2 SDs. Statistically significant values: *p<0.05; ***p<0.001. dpi, days postinoculation; OD, optical density.

At 14 dpi, infected chicks had increased IgY antibody levels in serum, demonstrating an antibody response to PDCoV (Figure 3, panel A), but sentinel chicks did not have antibody levels demonstrating exposure to PDCoV (Figure 3, panel B). Serum samples from poults exhibited a similar range of IgY values. The average IgY values were much higher in infected birds at 14 dpi compared with infected birds at 2 dpi and uninfected birds (Figure 3, panel C). The IgY greatly increased in sentinel poults at 14 dpi compared with IgY values at 2 dpi, but were still below the cutoff value (Figure 3, panel D).

### rRT-PCR on Samples from Chicks

**Figure 4:**
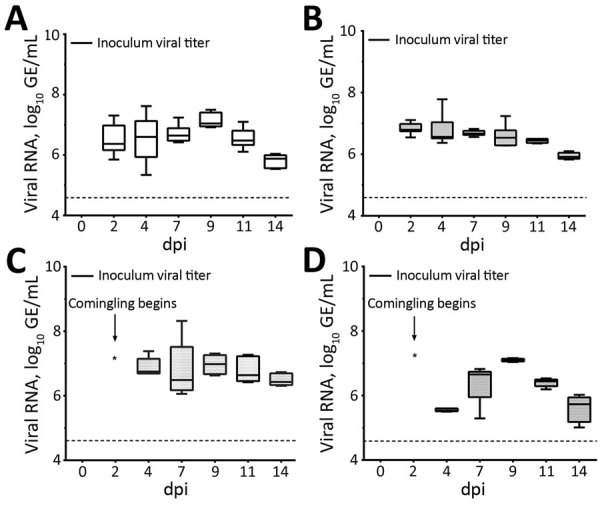
Porcine deltacoronavirus (PDCoV) viral RNA shedding patterns in samples collected from cloaca (A) and trachea (B) of infected and uninfected chicks, and from cloaca (C) and trachea (D) of sentinel chicks in a study of infection and transmission of porcine deltacoronavirus in poultry. Inoculum viral titer represents the genomic equivalent (GE) of inoculum administered at onset, 9.71 log_10_ GE/mL. Boxplots represent distribution of values; tops and bottoms of boxes represent 10%–90% range of values; horizontal lines within boxes indicate medians; error bars represent SEM. Dashed line indicates detection limit of 4.6 log_10_ GE/mL of PDCoV in samples. dpi, days postinoculation; GE, genomic equivalent.

All experimentally infected chicks rapidly shed detectable viral RNA postinoculation, and viral RNA titers remained relatively constant through 11 dpi (Figure 4, panels A, B). Viral RNA from cloacal swabs reached 6.52 log_10_ GE/mL by 2 dpi and remained >6.5 log_10_ GE/mL until 11 dpi, when levels at 7.14 log_10_ GE/mL at 9 dpi, then decreased to 5.82 log_10_ GE/mL at 14 dpi. Despite an absence of noticeable respiratory signs, tracheal swab specimens also showed high levels of PDCoV RNA throughout the study (Figure 4, panel B). PDCoV spread rapidly from infected to naive birds, and all 5 sentinel chicks became positive for PDCoV RNA in both tracheal and cloacal swabs within 2 days of comingling with infected birds (Figure 4, panels C, D).

**Figure 5:**
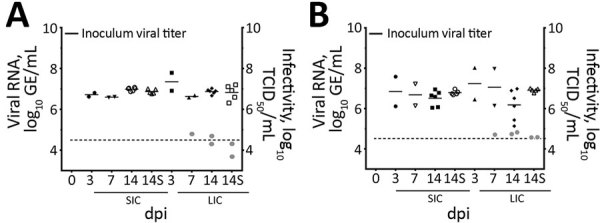
Viral RNA titers and infectivity of intestinal contents of (A) chicks and (B) poults in a study of infection and transmission of porcine deltacoronavirus in poultry. Inoculum viral titer represents the genomic equivalent (GE) of inoculum administered at onset, 9.71 log_10_ GE/mL. Gray dots represent infectivity at 7 and 14 dpi, expressed in log_10_ TCID_50_/mL, as indicated on the right *y*-axis. Dashed line indicates detection limit of 4.6 log_10_ GE/mL of PDCoV in samples. Shapes represent individual birds necropsied at each time point. Solid bars represent the mean. dpi, days postinoculation; GE, genomic equivalent; LIC, large intestine contents; S, sentinel birds necropsied; SIC, small intestine contents; TCID_50_, 50% tissue culture infectious dose.

We calculated titers and viral RNA loads in SIC and LIC from infected and sentinel chicks at 3, 7, and 14 dpi (Figure 5). We used RNA isolated at 14 dpi from SIC of 1 infected and 1 sentinel bird to amplify an ≈1,300-bp portion of the nucleocapsid (N) gene of PDCoV, then gel extracted and sequenced the resulting product. The samples sequenced had >99% identity with the original inoculum, Ohio FD22 strain of PDCoV. We tested infectivity of intestinal contents of infected and sentinel chicks by using TCID_50_ assay at 7 and 14 dpi (Figure 5, panel A).

### rRT-PCR on Samples from Poults

**Figure 6:**
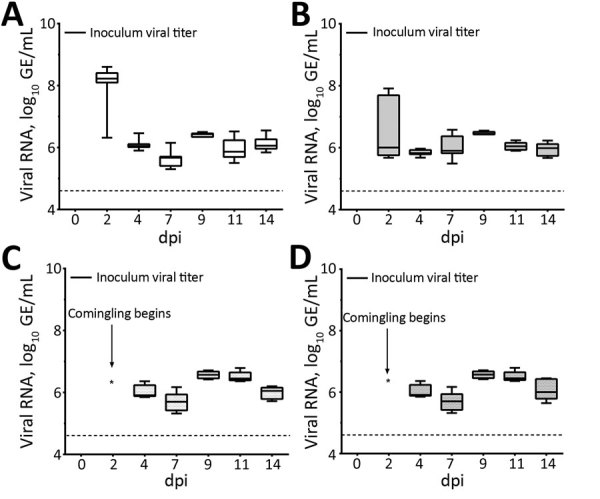
Porcine deltacoronavirus (PDCoV) viral RNA shedding patterns in samples collected from cloaca (A) and trachea (B) of infected and uninfected poults and from cloaca (C) and trachea (D) of sentinel poults in a study of infection and transmission of porcine deltacoronavirus in poultry. Inoculum viral titer represents the genomic equivalent (GE) of inoculum administered at onset, 9.71 log_10_ GE/mL. Box plots represent distribution of values; tops and bottoms of boxes represent 10%–90% range of values; horizontal lines within boxes indicate medians; error bars represent SEM. Dashed line indicates detection limit of 4.6 log_10_ GE/mL of PDCoV in samples. dpi, days postinoculation.

Similar to the results from chicks, results for infected poults showed all had high levels of PDCoV RNA in cloacal and tracheal swabs through 14 dpi (Figure 6, panels A, B). Poults appeared to have higher initial viral loads, averaging 8.07 log_10_ GE/mL by 2 dpi, decreasing to ≈6 log_10_ GE/mL at 4 dpi, and persisting through 14 dpi (Figure 6, panel A). Naive birds also were susceptible to infection, and cloacal and tracheal swab specimens from all sentinel poults were positive for PDCoV RNA within 2 days after comingling with infected poults (Figure 6, panels C, D, E). We calculated titers and viral RNA loads in SIC and LIC from infected and sentinel poults at 3, 7, and 14 dpi (Figure 5, panel B). We tested infectivity of intestinal contents of infected and sentinel poults by using TCID_50_ assay at 7 and 14 dpi (Figure 5, panel B).

We isolated viral RNA from the SIC of 1 infected and 1 sentinel bird at 14 dpi and used it to amplify an ≈1,300-bp portion of the N gene of PDCoV, then gel extracted and sequenced the resulting product. As we noted in chicks, the samples sequenced had >99% identity with the original inoculum, the Ohio FD22 strain of PDCoV.

## Discussion

Emerging viruses in at least 2 genera of porcine CoVs have exhibited increased propensity for interspecies transmission (*2*,*29*). Porcine APN was identified as a major cell entry receptor for PDCoV (*19*,*30*). APN is a protein that exhibits enzymatic activity, peptide processing, cholesterol uptake, and chemotaxis to cell signaling and cell adhesion (*31*). APN is widely distributed and highly conserved in amino acid sequences across species of the Animalia kingdom (*19*) and is expressed in a wide range of tissues, including epithelial cells of the kidneys (*31*), respiratory tract (*32*,*33*), and gastrointestinal tract (*34*).

Our data suggest that chicks and poults are susceptible to infection with PDCoV. In addition, the rapid transmission of PDCoV to the sentinel birds that comingled with infected birds demonstrates that the virus could spread easily. The length of our pilot study did not allow us to determine how long the chicks and poults would be affected by PDCoV or how long they might shed viral RNA. The chicks appeared to recover more rapidly than poults; clinical signs diminished or were completely absent by 14 dpi. However, chicks still were shedding low viral RNA titers at 14 dpi. Poults did not recover by the end of the study and still exhibited gross pathology and mild to moderate diarrhea. PDCoV RNA shedding titers were higher in poults than in the chicks. Cloacal shedding titers in chicks peaked at 9 dpi and then decreased. In poults, cloacal viral RNA shedding titers were multiphasic, peaking at 2 dpi, with additional smaller peaks at 9 and 14 dpi. The rapid onset of viral RNA shedding correlates with previous in vitro data in which the PDCoV S1 domain bound most efficiently to APN of galline origin (*19*) and cytopathic effects were observed more rapidly in leghorn male hepatoma and DF1 chicken cell lines compared with swine testicular cells (S.P. Kenney, unpub. data).

ELISA results showed that both chicks and poults developed PDCoV antibodies by 14 dpi. Pig infection dynamics have demonstrated a similar serum neutralizing antibody titer increase at 7–14 days (*12*,*23*). Sentinel birds had low or undetectable antibody responses compared with experimentally challenged birds, likely because of the passive infection method and because less time passed between exposure to the virus and the end of the study.

Recent studies demonstrated that PDCoV can infect and kill cells of other species through APN receptors (*19*,*35*). PDCoV has been reported to infect commercial chickens in vivo (*36*). The differences in susceptibility to PDCoV infection between chicks and poults we observed could be related to differences in APN expression levels between the species. 

The true incidence rates for PDCoV infection, natural host range, reservoirs, and routes of transmission are still relatively unknown, and no plans for vaccine development have been reported (*37*). DCoV RNA has been detected in fecal samples from wild birds (*38*,*39*), Chinese ferret badgers (*Melogale moschata*), and leopard cats (*40*). In addition to swine, calves have been shown by experimental testing to be susceptible to PDCoV infection (*41*). These data, coupled with the PDCoV binding receptor APN being conserved across many species, suggest that the host range for PDCoV is broader than initially expected (*19*). The close sequence homology between DCoV isolates from mammalian and wild bird species implies a transmission cycle in which PDCoV regularly crosses from wild birds and mammals into animal production systems, including the swine and poultry industries. More epidemiologic data are required to understand the full extent to which DCoVs are threatening food production systems and whether they pose a direct threat to human health. 

Our results are consistent with the likelihood that avian species act as potential passthrough or intermediate hosts for PDCoV. In vivo confirmation of avian susceptibility to PDCoV suggests that in vitro data implicating human susceptibility should be evaluated further. Research regarding how PDCoV is adapting and mutating in different species and whether it infects humans is critical to determining if PDCoV poses a pandemic health risk to commercial poultry or humans.
